# Altered Cortical Gyrification Morphology in Nonsucidal Self-injury

**DOI:** 10.1192/j.eurpsy.2024.1285

**Published:** 2024-08-27

**Authors:** S. Choi, H. Moon, J.-W. Hur

**Affiliations:** Department of Psychology, Korea University, Seoul, Korea, Republic Of

## Abstract

**Introduction:**

Nonsuicidal self-injury (NSSI) is defined as deliberate and direct damage to one’s body tissues without any suicidal intent. NSSI is now recognized as a major risk factor for suicide and is prevalent among adolescents, with prevalence rates ranging from 7.5% to 46.5%, leading to increased interest in the pathophysiology of NSSI. This study aimed to examine cortical gyrification morphology, a neurobiological index of cortical folding and patterning, among unmedicated individuals with NSSI, which is prevalent in adolescents and young adults.

**Objectives:**

The main objective of this study is to compare cortical morphological abnormalities between individuals with NSSI and controls in terms of the local gyrification index (LGI), the ratio of the smooth cortical surface area at each vertex to the corresponding sulcal folds. In addition, we hypothesized that the LGI, a stable neurodevelopmental marker of cortical and subcortical circuit intergrity, would correlate with clinical measures in youth with NSSI.

**Methods:**

A total of 101 individuals with NSSI and 100 age-, gender-, and handedness-matched controls completed self-report questionnaires and structural magnetic resonance imaging (MRI) data were acquired on a 3T Siemens scanner. A surface-based analysis was conducted using the Computational Anatomy Toolbox (CAT12) in Statistical Parametric Mapping (SPM12). Partial correlation analysis was also performed using R software to investigate the association between the LGI values extracted from the region of interest (ROI) and clinical symptoms, including depression, anxiety, emotion dysregulation, and anhedonia in individuals with NSSI.

**Results:**

Individuals with NSSI showed significantly increased LGI in the right insula sulcus and left superior temporal sulcus (STS), along with decreased LGI in the right calcarine and left superior parietal sulcus (SPS), compared to controls (5000 permutation correction, threshold-free cluster enhancement with a threshold of *p* < .05). In addition, higher LGI in left STS was correlated with greater scores of the Beck Anxiety Inventory (*r* = 0.22*, p* < .05) and of the Impulse Control Difficulties subscale of the Difficulties in Emotion Regulation Scale (*r* = 0.34*, p* < .001). Conversely, reduced LGI of the right calcarine was associated with a higher score on the Anhedonia subscale of the Beck Depression Inventory (*r* = -0.23*, p* < .05) within individuals with NSSI.

**Conclusions:**

This study identified hypergyria in the right insular and left STS and hypogyria in the right calcarine and left SPS in individuals with NSSI. The former pattern was associated with anxiety and impulse control difficulties, and the latter was with anhedonia. This study is the first to alter distinct neurodevelopmental patterns of local gyrification and their correlations with clinical manifestations in individuals with NSSI.

**Disclosure of Interest:**

None Declared

